# Resonant optical Stark effect in monolayer WS_2_

**DOI:** 10.1038/s41467-019-13501-x

**Published:** 2019-12-05

**Authors:** Paul D. Cunningham, Aubrey T. Hanbicki, Thomas L. Reinecke, Kathleen M. McCreary, Berend T. Jonker

**Affiliations:** 1 U.S. Naval Research Laboratory, 4555 Overlook Avenue SW, Washington, DC 20375 USA; 2 Present Address: Laboratory for Physical Sciences, University of Maryland, 8050 Greenmead Drive, College Park, MD 20740 USA

**Keywords:** Electronic properties and materials, Two-dimensional materials, Ultrafast photonics, Quantum optics

## Abstract

Breaking the valley degeneracy in monolayer transition metal dichalcogenides through the valley-selective optical Stark effect (OSE) can be exploited for classical and quantum valleytronic operations such as coherent manipulation of valley superposition states. The strong light-matter interactions responsible for the OSE have historically been described by a two-level dressed-atom model, which assumes noninteracting particles. Here we experimentally show that this model, which works well in semiconductors far from resonance, does not apply for excitation near the exciton resonance in monolayer WS_2_. Instead, we show that an excitonic model of the OSE, which includes many-body Coulomb interactions, is required. We confirm the prediction from this theory that many-body effects between virtual excitons produce a dominant blue-shift for photoexcitation detuned from resonance by less than the exciton binding energy. As such, we suggest that our findings are general to low-dimensional semiconductors that support bound excitons and other many-body Coulomb interactions.

## Abstract (web summary)

Many-body interactions have important consequences for the optoelectronic properties of 2D materials. Here, the authors report on how many-body interactions affect the behavior of the valley-selective optical Stark effect for excitation near the A-exciton resonance in monolayer WS_2_.

## Introduction

Broken inversion symmetry gives rise to valley selective electron populations and valley pseudospin in monolayer transition metal dichalcogenides (TMDs)^1,2^. The ability to control this valley degree of freedom has implications for both spintronics and the emerging field of valleytronics. Through the valley-selective optical Stark effect (OSE), intense circularly polarized light can break the valley degeneracy and allow selective tuning of the exciton energy levels in each valley^3,4^, which would otherwise require large magnetic fields through the valley Zeeman effect^5,6^. This process can be exploited for coherent manipulation of valley-superposition states for quantum information^7,8^, and may allow for high-speed valley-switching or the generation of polarization-entangled photon pairs^9^. Additionally, the OSE can provide insight into other strong light−matter interactions, such as exciton-polariton formation, that emerge due to near-field enhancement in cavities^10–12^ and around plasmonic nanostructures^13^.

The OSE is a coherent interaction between electronic energy levels in matter and a pump photon field that results in a shift of the transition energies^14^. This coherent interaction is enhanced by the strong light−matter coupling found in quantum wells^15^, quantum dots^16^, atomically thin semiconductors^3,17^, and recently discovered in bulk organometallic perovskite films^18^. Optical selection rules cause these interactions to be dependent on the polarization of the light field. In monolayer transition metal dichalcogenides, this manifests as a valley-selective OSE due to the opposite chiral optical selection rules for the degenerate *K*- and *K*′-valleys.

Typically, the OSE is accessed through a nonresonant photon field to avoid the dissipation and incoherent effects associated with excited state populations. Far from resonance, higher-lying excited states are typically unimportant and the material is treated as a two-level system. Traditionally, the OSE is understood using the dressed-atom picture^19^. In this picture, the coherent interaction of the ground and excited states with the light field gives rise to so-called Floquet states. These are hybrid light−matter states consisting of the electronic energy levels, e.g. ground and exciton states |*g*〉 and |*x*〉 respectively, dressed by a photon, e.g. to form |*g* ± *hυ*〉 and |*x* ± *hυ*〉 respectively. These short-lived Floquet states interact with the equilibrium matter states via the Coulomb potential and hybridize, leading to energy-level repulsion. The underlying physics of this process is similar to the formation of molecular excitons^20^ and is analogous to avoided crossing behavior between light and matter states through strong coupling in photonic cavities^21^. Though this picture treats the excited states as noninteracting particles, it has been applied to low-dimensional semiconductors where reduced electrostatic screening produces strong many-body interactions. For example, many-body Coulomb interactions give rise to observations of biexciton formation^22^, many-particle complexes^23–25^, efficient Auger recombination^26–28^, enhanced bandgap renormalization^29–31^, and the formation of electron-hole liquids^32^ in monolayer TMDs. Such many-body effects require a theoretical description that goes beyond the dressed-atom picture in order to understand the OSE in the strongly interacting limit.

Here we report the behavior of the valley-selective optical Stark effect for excitation near the A-exciton exciton resonance in monolayer WS_2_. We observe that both below- and above-resonance pumping with positive helicity pulses (*σ*^+^) produces a blue-shift of the *K*-valley in monolayer WS_2_. By contrast, no change is observed in the *K*-valley energy when pumped with negative helicity pulses (*σ*^−^). In that case, a photo-induced absorption associated with coherent creation of intervalley biexcitons is observed. While these observations are indicative of a valley-selective optical Stark effect, only the below-resonance behavior is qualitatively consistent with the dressed-atom picture. We show that an understanding of the OSE near-resonance requires accounting for many-body interactions between excitons, as proposed by Schmitt-Rink and Chemla^33^, Combescot and Combescot^34^, and Zimmerman^35^. The observed blue-shift arises from repulsion between coherently created virtual excitons, which dominates when the applied photon field is detuned from resonance by less than the exciton binding energy. These interactions contribute significantly to the light−matter interactions in low-dimensional semiconductors, like TMDs, that support bound excitons.

## Results

### Valley-selective optical Stark effect in monolayer WS_2_

**Fig. 1:**
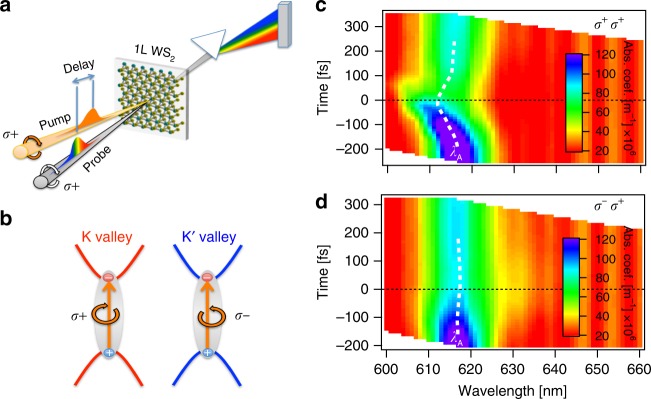
The valley-selective optical Stark effect in monolayer WS_2_. **a** Schematic of the valley-resolved transient absorption experiment. **b** The *K*- and *K*′- valleys are populated by right-hand (*σ*^+^) and left-hand (*σ*^−^) circularly polarized light due to chiral selection rules. **c** Summary of time-dependent, room temperature, absorption spectra of monolayer 1L WS_2_ recorded with a *σ*^+^ white-light probe upon 610 nm excitation with co- (*σ*^+^*σ*^+^) and **d** cross-circularly polarized (*σ*^−^*σ*^+^) light. The center wavelength of the A-exciton resonance (*λ*_A_) is indicated with a dashed white light. Time zero indicates when the pump and probe pulse are temporally overlapped.

We monitored the photo-induced changes in the *K*- and *K*′-valleys in monolayer WS_2_ using transient absorption (TA) spectroscopy, schematically shown in Fig. 1a. To exploit the chiral optical selection rules in WS_2_^1^, shown in Fig. 1b, we use both a circularly polarized pump excitation pulse to drive dynamics in a particular valley as well as a circularly polarized white-light probe pulse to observe the changes induced in either valley. This technique allows for the time-dependent evolution of the absorption spectra of each valley to be directly measured just after photoexcitation. In this way, we can observe reduced absorption associated with state filling^28^, photo-induced absorption associated with excited state transitions, changes in absorption line width, and spectral shifts in energy levels associated with the OSE^4^, dynamic Coulomb screening^31,36^, and lattice heating^37^. While time-resolved Kerr rotation is a more sensitive technique than TA and is capable of resolving <1 meV spectral shifts^38^, for excitation near resonance we expect large optical Stark shifts that can be easily observed with TA.

Figure 1c, d shows a compilation of the room temperature valley-resolved TA spectra in monolayer WS_2_ for a photoexcitation wavelength of 610 nm, which is just above the A-exciton resonance located at 616 ± 1 nm. When both pump and probe pulses are right-hand circularly polarized (*σ*^+^*σ*^+^), i.e. are cocircularly polarized, we observe an instrument limited blue-shift of the absorption spectrum of the *K*-valley, as shown in Fig. 1c. This blue-shift occurs only at time zero, when pump and probe are temporally overlapped. This indicates that the observed shift arises from coherent light−matter interactions between the pump pulse and the sample rather than incoherent processes associated with photoexcited excitons or charge carriers. By contrast, no significant shift is observed in the *K*-valley for cross-circular polarizations (*σ*^−^*σ*^+^), i.e. when a left-hand circularly polarized pump coherently drives the *K*′-valley, as shown in Fig. 1d. This behavior is consistent with a valley-selective OSE in WS_2_^4^. The applied field of the *σ*^+^ (*σ*^−^) pulses couples selectively with the *K*− (*K*′−) valley. Driving the *K*′-valley has no effect on the *K*-valley energies. Therefore, this coherent light−matter interaction is well isolated to a single valley so that we only observe an optical Stark shift for cocircularly polarized light. The small residual blue-shift (~1 meV) that persists for picoseconds after photoexcitation is associated with bandgap renormalization from the photoexcited exciton population^31^, which is an incoherent process unrelated to the OSE and occurs on a significantly slower timescale.

### Coherent intervalley biexciton generation in monolayer WS_2_

**Fig. 2:**
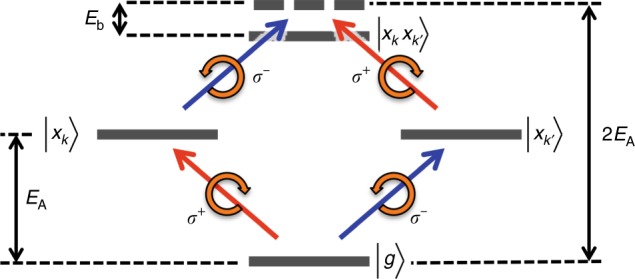
Intervalley biexciton formation from sequential photon absorption. A right-hand (*σ*^+^) or left-hand (*σ*^−^) circularly polarized pulse raises the system from the ground state, |*g*〉, to populate either the *K*- or *K*′-valley with an exciton, |*x*_*K*_〉 or |*x*_*K*′_〉 respectively. A second photon of opposite chirality can then transition the system from the valley exciton to the intervalley biexciton state via the process $|x_{K^{\prime} }\rangle \to |x_{K^{\prime} }x_K\rangle$ or $|x_K\rangle \to \,|x_Kx_{K^{\prime} }\rangle$. This bound intervalley biexciton is reduced by its binding energy, *E*_b_, to below twice the exciton energy, *E*_A_.

It is important to note that for cross-circular polarizations (*σ*^−^*σ*^+^), a photo-induced absorption band appears below the A-exciton resonance in Fig. 1d, centered at 631 ± 2 nm; see Supplementary Note 1 and Supplementary Fig. 1 for details. Strong Coulomb interactions in TMDs produce many-body interactions that give rise to the formation of stable biexcitons^22^. Owing to the degenerate *K*- and *K*′-valleys as well as the small spin-orbit splitting of the conduction band, there are a number of possible biexcitons in TMDs: intra- and intervalley, charged and neutral, and those consisting of combinations of bright and dark excitons. The optical selection rules tell us that a *σ*^−^*σ*^+^ pulse progression will not create intravalley biexcitons, which would also have a higher energy due to Pauli repulsion of the two holes in the valence band. While bright-dark intervalley biexcitons form preferentially in tungsten-based TMDs due to the reservoir of energetically favorable dark excitons^39^, they are spin and momentum forbidden and will not be directly photoexcited. Instead, a scattering process is required to create the dark exciton such that bright-dark intervalley biexcitons cannot be created coherently. We therefore tentatively assign the photo-induced absorption feature to the coherent creation of intervalley biexcitons. Figure 2 shows schematically how the neutral intervalley biexciton is generated. The σ- pump photon creates an exciton in the *K*′-valley. The subsequent *σ*^+^ probe photon can then access the |*x*_*K*′_〉 → |*x*_*K*_, *x*_*K*′_〉 transition. This process is expected to be general to the monolayer TMD semiconductors. The photo-induced absorption feature is located approximately 48 ± 7 meV below the A-exciton, consistent with recent measurements of biexciton emission in monolayer WS_2_^39^. Because this absorption band is also near the emission band reported for charged biexcitons in the tungsten-based TMDs^23–25^, we cannot exclude their participation in this photo-induced absorption. It should be noted that in those measurements the TMDs were encapsulated in hBN and graphene, both of which have been shown to reduce exciton binding energies through screening of Coulomb interactions^40^. It is likely that such an effect would reduce the binding energy of multiparticle complexes as well, so that we may expect the observed binding energies to be larger for our case of monolayer WS_2_ on fused silica. Also, since charged biexcitons are exciton-trion complexes, we do not expect their contribution to be large due to the lack of trionic character of the A-exciton absorption at room temperature. Additionally, the trion formation time is too slow, >1 ps^41,42^, for trions to have a large contribution to the observed ultrafast photo-induced absorption. It is worth noting that we are able to identify features associated with trions and those potentially arising from charged biexcitons in WS_2_ at low temperature; see Supplementary Note 2 and Supplementary Figs. 2 and 3 for details. The relatively short timescale over which coherent biexciton creation can proceed may be related to the short valley coherence times for the A-exciton in TMDs at room temperature^8^. While this induced absorption feature is a coherent process, it is unrelated to the OSE.

### Dressed-atom model of the optical Stark effect

**Fig. 3:**
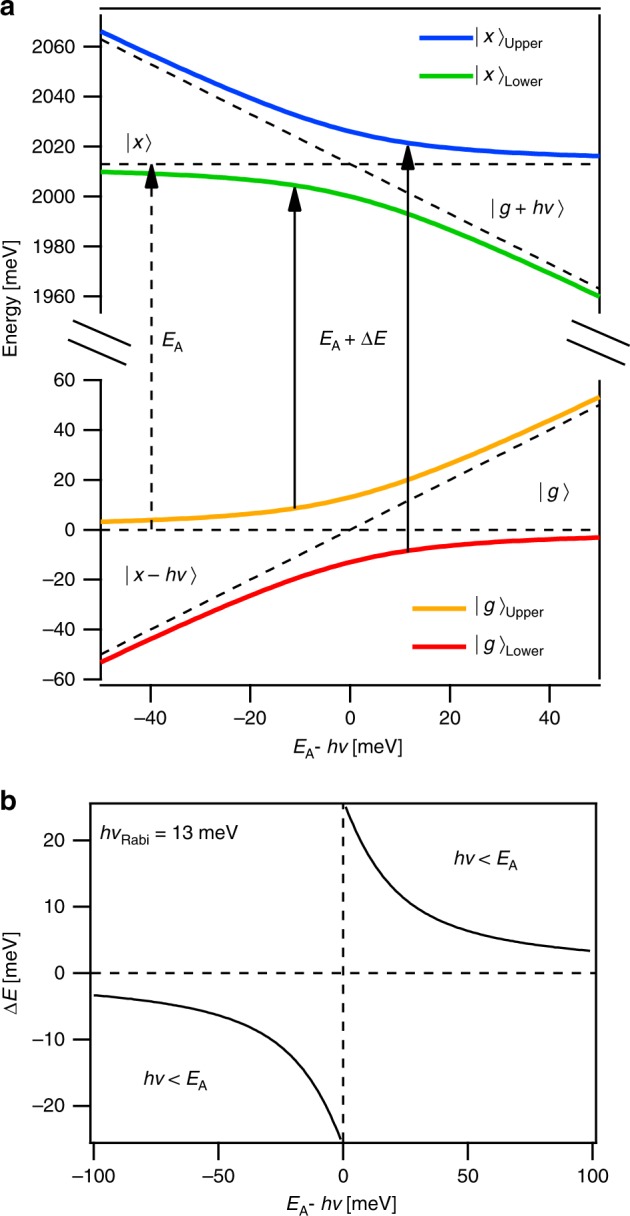
Two-level dressed-atom model of the optical Stark effect. **a** Energy-level diagram showing the equilibrium ground, |*g*〉, and exciton, |*x*〉 states (dashed horizontal lines) that mix with the photon-dressed states, |*g* + *hυ*〉 and |*x* − *hυ*〉 (dashed diagonal lines), and hybridize. Energy-level repulsion results in upper and lower exciton-polariton bands, e.g. |*x*〉_upper_ and |*x*〉_lower_, with optical transitions shifted by, *∆E*. **b** The optical Stark shift predicted by the two-level dressed-atom model, calculated from Eq. (3), for *hν*_R_ = 13 meV i.e. a Rabi frequency of 105 cm^−1^.

The valley-selective OSE in TMD monolayers has been previously described in the context of a dressed-atom picture, where the exciton states are treated as noninteracting atom-like states^3,4^. In this semi-classical theory, the ground state, |*g*〉, and A-exciton, |*x*〉, hybridize with the Floquet (i.e. photon-dressed) states, |*g* + *hυ*〉 and |*x*  − *hυ*〉 through light−matter coupling. This is shown schematically in Fig. 3. In this simple two-level model, we can use the equilibrium and photon-dressed states as a basis set to construct the effective Hamiltonian for the light−matter interaction,

1 $$H_{{\mathrm{{eff}}}} = \left( {\begin{array}{*{20}{c}} 0 & V & 0 & 0 \\ V & \Delta & 0 & 0 \\ 0 & 0 & {E_{\mathrm{A}} - \Delta } & V \\ 0 & 0 & V & {E_{\mathrm{A}}} \end{array}} \right).$$

Here the light−matter coupling, *V*, can be expressed as $V = M_{gx}{\cal{E}}$, where ${\cal{E}}$ is the applied photon field and *M*_*gx*_ corresponds to the polarization matrix element between the ground state and A-exciton. It should be noted that the Rabi frequency associated with the light−matter coupling is $\nu _R = M_{gx}{\cal{E}}/h$ so that the light−matter coupling can also be expressed as $V = h\nu _{\mathrm{R}}$. For simplicity we also introduce the photon energy detuning, $\Delta = E_{\mathrm{A}} - h\upsilon$. Diagonalizing the Hamiltonian yields energy eigenstates

2 $$\begin{array}{*{20}{c}} {E_{\left| x \right\rangle _{{\mathrm{{lower}}}}^{{\mathrm{{upper}}}}} = E_{\left| x \right\rangle } - \frac{{\mathrm{\Delta }}}{2} \pm \frac{1}{2}\sqrt {{\mathrm{\Delta }}^2 + 4V^2} } \\ {E_{\left| g \right\rangle _{{\mathrm{{lower}}}}^{{\mathrm{{upper}}}}} = E_{\left| g \right\rangle } + \frac{{\mathrm{\Delta }}}{2} \pm \frac{1}{2}\sqrt {{\mathrm{\Delta }}^2 + 4V^2} } \end{array},$$

where *E*_|*g*〉_ = 0 and *E*_|*x*〉_ = *E*_A_. As expected, Eq. (2) shows that the quantum mechanical coupling between the equilibrium matter and photon-dressed states leads to energy-level repulsion and results in the formation of upper and lower exciton-polariton bands. The optical absorption spectrum can be computed as the transitions between these eigenstates. For positive detunings, where the photon energy is below resonance, the typical blue-shifted absorption band due to the OSE is the transition between the lower ground-state band, |*g*〉_lower_, and the upper exciton band, |*x*〉_upper_. The energy-shift of the exciton resonance is given by

3 $$\Delta E = - {\mathrm{\Delta }} \pm \sqrt {{\mathrm{\Delta }}^2 + 4V^2} ,$$

where $\sqrt {{\mathrm{\Delta }}^2 + 4V^2}$ is the generalized Rabi frequency. Far from resonance, the weak field approximation, $M_{gx}^2{\cal{E}}^2/\Delta \ll 1,$typically applies and the energy-shift reduces to the familiar expression

4 $$\Delta E \cong 2\frac{{M_{gx}^2{\cal{E}}^2}}{\Delta }.$$

Importantly, the two-level dressed-atom model predicts that for increasing photon energies, the OSE energy-shift will change signs from a blue-shift for *hυ* < *E*_A_ to a red-shift for *hυ* > *E*_A_. Experimentally, as is shown in Fig. 1c, we instead observe that the blue-shift persists even for photon energies above the A-exciton resonance. Extending the two-level dressed-atom model to include biexciton levels^34,43,44^ does not describe our observations.

### Excitonic model of the optical Stark effect

The dressed-atom model has successfully described semiconductors excited far from resonance^15^, where the effective Rabi frequency is larger than the inverse time required to form an exciton. In that limit, Coulomb interactions are unimportant on the timescale of the light−matter interaction. However, near resonance these interactions play an important role. Schmitt-Rink and Chemla^33^ developed a theoretical description of the excitonic OSE in semiconductors that includes these many-body Coulomb interactions. There a two-level system Hamiltonian, similar to Eq. (1), is used with the addition of an interaction potential that depends on the Coulomb coupling. Optical excitation coherently creates a virtual exciton population, present only during the duration of the pulse. This coherent nature implies that relaxation processes, e.g. electron−phonon interactions, are not present for the virtual exciton population. The many-body interactions between virtual excitons are treated within a Hartree-Fock approximation. The optical Stark shift is obtained by evaluating the coherent polarization induced by the pump photon field and felt by a weak probe field. It gives the optical Stark shift in terms of contributions from exciton−photon interactions and exciton−exciton interactions. For simplicity, the exciton spectrum is approximated by the lowest lying 1S exciton, which describes the overall behavior well^35^. Full details of the derivation of this theory, hereafter referred to as the excitonic OSE model, are given in the review by Schmitt-Rink et al.^45^. The optical Stark shift can be expressed for a 2D system in the low excitation limit, $Na_0^2 \ll 1$, as

5 $$\Delta E \cong 2\frac{{M_{gx}^2{\cal{E}}^2}}{\Delta }\left( {\frac{{\left| {\phi _{1s}\left( {r = 0} \right)} \right|^2}}{{N_s^{{\mathrm{{PSF}}}}}}} \right) + 8\pi Na_0^2\left( {1 - \frac{{315\pi ^2}}{{4096}}} \right)E_{\mathrm{b}},$$

where *N* is the virtual exciton density and *a*_0_ is the exciton Bohr radius. For a Bohr radius of 1 nm, typical of TMDs^46^, this approximation should hold for virtual exciton densities *N* ≪ 1.1 × 10^−14^ cm^−2^. The first term in Eq. (5) arises from the exciton−photon interaction, and is identical to the dressed-atom result in Eq. (4) renormalized by a multiplicative factor accounting for excitonic effects. The numerator, $\left| {\phi _{{\mathrm{{1s}}}}\left( {r = 0} \right)} \right|^2$, is the 1S exciton wave function and reflects the fact that excitons are built from linear combinations of atomic Bloch states. The denominator contains the saturation density due to phase-space filling, which is approximated as $N_s^{{\mathrm{{PSF}}}} \approx 1/2\pi a_0^2$ when thermal energy is much less than the exciton binding energy, *E*_b_. Phase-space filling gives rise to a decrease in the 1S exciton oscillator strength and saturation of the optical Stark shift for high fields, which have previously been experimentally confirmed^47^. In two dimensions, the excitonic factor can be approximated as $\left| {\phi _{{\mathrm{{1s}}}}\left( {r = 0} \right)} \right|^2/N_s^{{\mathrm{{PSF}}}} \approx 16/7$. The second term in Eq. (5) is due to exciton-exciton interactions for 1 S excitons in a 2D system. The virtual exciton density can be approximated as $N \approx 2M_{gx}^2{\cal{E}}^2\left| {\phi _{1s}\left( {r = 0} \right)} \right|^2/\Delta ^2$^45^. Then Eq. (5) becomes

6 $${\mathrm{\Delta }}E \approx 2\frac{{M_{gx}^2{\cal{E}}^2}}{{\mathrm{\Delta }}}\left( {\frac{{16}}{7}} \right) + 8\frac{{M_{gx}^2{\cal{E}}^2}}{{{\mathrm{\Delta }}^2}}\left( {\frac{{16}}{7}} \right)\left( {1 - \frac{{315\pi ^2}}{{4096}}} \right)E_b.$$

**Fig. 4:**
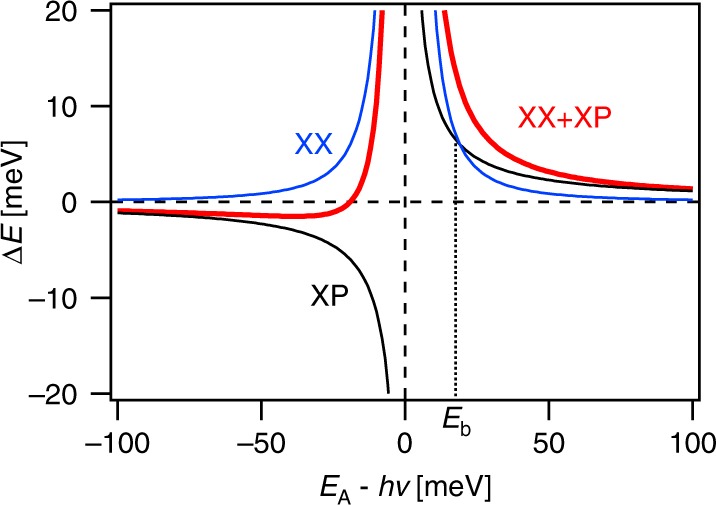
Excitonic model of the optical Stark effect. Example calculation of the optical Stark shift predicted by the excitonic OSE model^33^ in Eq. (6) (red), for *hν*_R_ = 5 meV, i.e. a Rabi frequency of 40 cm^−1^, and an exciton binding energy *E*_b_ = 20 meV. The exciton−photon (XP, black) and exciton−exciton (XX, blue) contributions are shown.

An independent derivation by Combescot and Combescot^34^ arrives at a similar expression for the optical Stark shift in the presence of Coulomb repulsion between virtual excitons. The two terms in Eq. (6) are shown in Fig. 4. The exciton−photon interaction produces a shift in the exciton energy that changes sign as the photon field is tuned through resonance, while the exciton−exciton interaction is repulsive and produces a blue-shift for all detunings. The exciton−exciton interaction dominates when the detuning from the exciton resonance is less than the exciton binding energy, Δ < *E*_b_. Numerical calculations of the optical Stark effect by Zimmerman^35^ that include the full spectrum of exciton states confirm that exciton−exciton repulsion, proportional to 1/Δ^2^, dominates for small detuning. Therefore, inclusion of such exciton−exciton interactions is important for understanding the OSE in low-dimensional semiconductors that support bound excitons, such as TMDs.

### Power dependence of the optical Stark effect

**Fig. 5:**
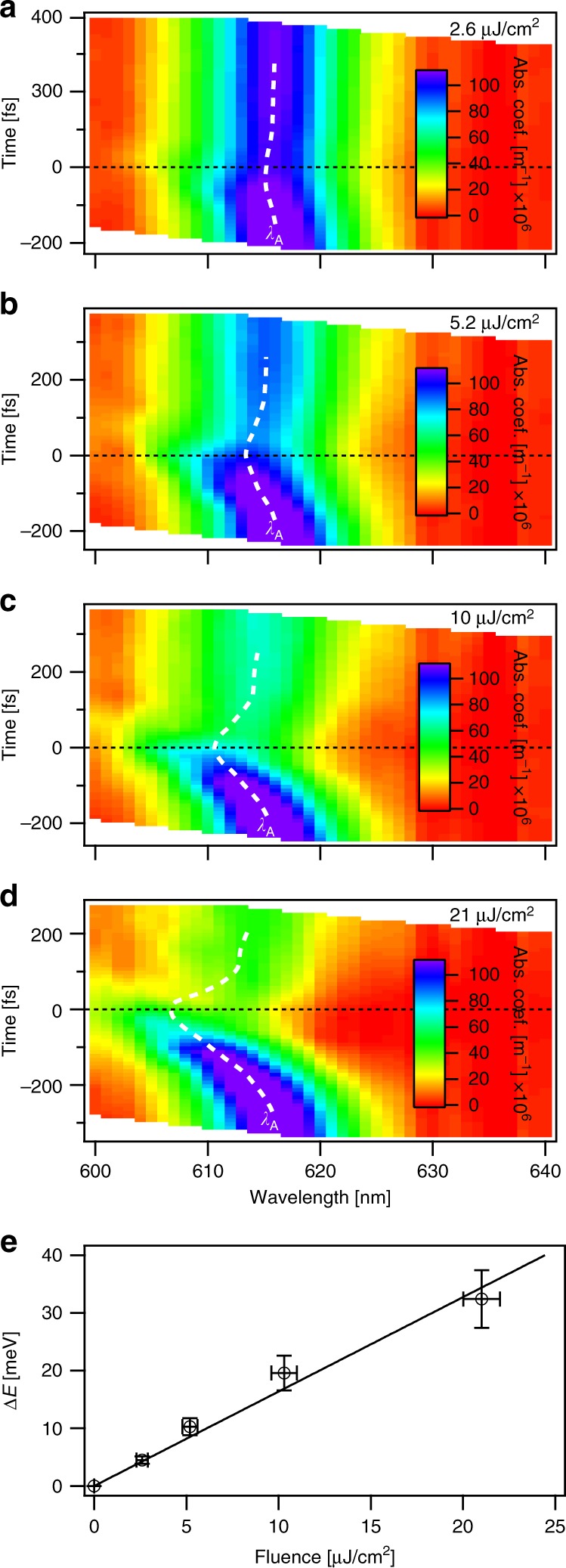
Power dependence of the optical Stark effect. Time-dependent absorption spectra of monolayer WS_2_ recorded for cocircularly polarized 610 nm pump pulses with incident fluences of **a** 2.6, **b** 5.2, **c** 10, and **d** 21 µJ/cm^2^. The center wavelength of the A-exciton resonance (*λ*_A_) is indicated with a dashed white line. Time zero indicates when the pump and probe pulse are temporally overlapped. **e** Shows the fluence dependence of the maximum optical Stark shift, *∆E*. The error bars in fluence represent the propagated uncertainty in pump power and beam diameter. The error bars in *∆E* represent the sum of uncertainties in center wavelength of the unperturbed A-exciton absorption and of the absorption at time zero. The solid line is a fit to Eq. (6) for 150 fs pulses, *M*_*gx*_ = 5.2 ± 0.4 D, *E*_b_ = 320 ± 40 meV, and a detuning of −23 meV.

To further understand the behavior of the OSE, we examined the pump power dependence. Figure 5 shows that for 610 nm excitation, the blue-shift observed for co-circular polarizations increases with pump power, here expressed as incident fluence. The corresponding incident optical fields span 17.2–138 MW/cm^2^, and the absorbed photon density range of 3.9 × 10^11^–3.1 × 10^12^ photons/cm^2^ is below the Mott density of 10^13^−10^14^ cm^−2^. The maximum photo-induced shifts were estimated by fitting the time-dependent absorption spectra with a Gaussian peak; see Supplementary Note 3 and Supplementary Fig. 4 for details. The observed optical Stark shift, Δ*E*, is well-described by the excitonic OSE model^33^ in Eq. (6) for *M*_*gx*_ = 5.2 ± 0.4 D. Here we have used the experimentally determined exciton binding energy, *E*_b_, of 320 ± 40 meV for WS_2_ on fused silica^31,48^. Note that the binding energy is located between estimates of the 3D exciton Rydberg, ${\mathrm{{Ry}}} = \left( {e^4\mu } \right)/\left( {2\hbar ^2\varepsilon ^2} \right) \approx 167\,{\mathrm{meV}}$, and 2D exciton Rydberg, 4Ry ≈ 668 meV^49^, which is consistent with quasi-two-dimensionality and with the Hamiltonian used in the excitonic OSE model. The estimated dipole moment is similar to previous estimations of *M*_*gx*_ = 6.7 D and 7.7 D from OSE^3^ and absorption^50^ measurements of monolayer WSe_2_, respectively. For the largest power density, which corresponds to *hν*_R_ ~ 3.5 ± 0.4 meV and a Rabi frequency of 28 ± 3 cm^−1^, we observe an optical Stark shift of 32 ± 5 meV, which is the largest optical Stark shift reported for a TMD monolayer. To achieve such a large spectral shift through the valley-Zeeman effect would require magnetic fields near 190 T^3^. Despite pumping so close to resonance, we do not observe any Autler−Townes-type splitting of the A-exciton absorption band^14^. Such splitting has recently been observed when accessing the OSE in MoSe_2_ by pumping the 1S-2P transitions in the mid-infrared^51^.

### Detuning dependence of the optical Stark effect

**Fig. 6:**
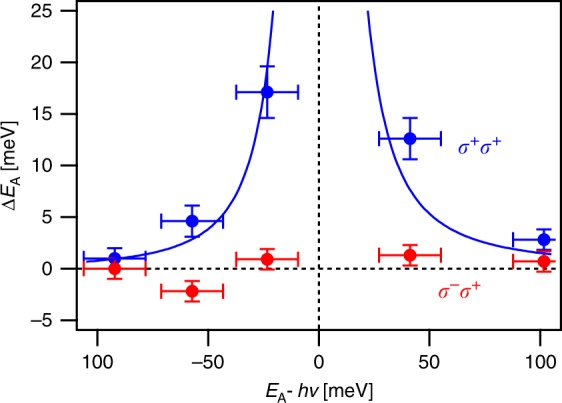
Optical Stark effect near A-exciton resonance in monolayer WS_2_. The maximum photo-induced shift of the A-exciton energy recorded for co- (blue) and cross-circularly polarized (red) pulses as a function of pump photon energy detuning. The pump power was kept constant throughout. The error bars in detuning represent the sum of the estimated bandwidth of a 150 fs Gaussian pulse (~12 meV) and uncertainties in the center wavelengths of both the pump pulse and the unperturbed A-exciton absorption. The error bars in *∆E* represent the sum of uncertainties in center wavelength of the unperturbed A-exciton absorption and of the absorption at time zero. The solid blue line is a fit to Eq. (6) for 150 fs 10 µJ/cm^2^ pulses, *E*_b_ = 320 meV, and *M*_*gx*_ = 6.1 ± 0.7 D.

We also examined the excitation wavelength dependence of the observed optical Stark shift for photoexcitation near the A-exciton resonance; see Supplementary Note 4 and Supplementary Fig. 5. Again, the maximum photo-induced shifts were estimated by fitting the time-dependent absorption spectra with a Gaussian peak (see Supplementary Note 3 and Supplementary Fig. 4 for details). Figure 6 shows the optical Stark shift observed for both co- and cross-circular polarizations as a function of pump detuning. Regardless of excitation wavelength, the observed Stark shift is nearly zero for cross-circular polarizations, which is consistent with the valley-selective OSE. Also, we do not observe the small Stark shifts predicted for cross-circular polarization when the pump photon energies are near the intervalley biexciton resonance^34^. Recent experimental observations of that effect in MoSe_2_ required narrow band pump excitation not available in the experiments presented here^44^. There, a ~2 meV optical Stark shift was observed for excitation at *hυ* = *E*_A_ − *E*_b_ with an optical field of 65 MW/cm^2^; the similarly small shift expected here for 10 µJ/cm^2^ may be too small for our resolution. For co-circular polarization, we observe the familiar blue-shift for excitation energy below the A-exciton resonance, consistent with the valley-selective OSE. As expected, the induced Stark shift becomes larger as the excitation energy approaches the A-exciton resonance. For excitation above resonance, we continue to see a blue-shift. This detuning-dependent behavior is consistent with the excitonic OSE model^33^, where repulsion among virtual excitons can produce a blue-shift for excitation above the exciton resonance^35^. The observed detuning dependence of the optical Stark shift, Δ*E*(Δ), is well-described by Eq. (6) for an energy density corresponding to 150 fs 10 µJ/cm^2^ pulses, *E*_b_ = 320 meV, and *M*_*gx*_ = 6.1 ± 0.7 D, consistent with *hν*_R_ of 2.9 ± 0.2 meV and a Rabi frequency of 22 ± 2 cm^−1^. The value of polarization matrix element *M*_*gx*_ is in agreement with the value estimated from power-dependent measurements in Fig. 5 as well as estimates in literature for monolayer WSe_2_^3^.

## Discussion

Our measurements show that the dressed-atom picture does not describe the OSE for near resonance excitation of monolayer WS_2_, where many-body Coulomb interactions become significant. In this regime, an excitonic OSE model, like that described by Schmitt-Rink and Chemla^33^, Combescot and Combescot^34^, and Zimmerman^35^, is required. For pump photons detuned from resonance by less than the exciton binding energy, exciton−exciton interactions that arise due to repulsion among coherently created virtual excitons dominate over the exciton−photon interactions that typically describe the light−matter interaction far from resonance. This produces an optical Stark shift that is larger than expected for below-resonance excitation. Additionally, and most strikingly, the exciton−exciton interaction produces a blue-shift for optical excitation above the exciton resonance. The measurements presented here are, to the best of our knowledge, the first experimental confirmation of this predicted above-resonance blue-shift and the dominance of exciton−exciton interactions. Our observations are likely general to TMDs and should apply to other low-dimensional semiconductors that exhibit strong many-body Coulomb interactions.

Our observations have consequences for predicting the outcome of other strong light−matter interactions in TMD monolayers. First, a band-inversion and resulting Floquet-driven chiral topological states have been predicted for above-resonance excitation based on application of the two-level dressed-atom model to TMD monolayers^4^. Since that model is not applicable, it may introduce doubt that such a band-inversion will occur, and may explain the absence of evidence for these topological edge states. Additionally, we expect that our observations may also affect the behavior of valley-polarized interlayer excitons in TMDs^52,53^. For example, optical excitation above the interlayer exciton resonance may not lead to the expected red-shift of both intravalley excitons in the individual monolayers due to repulsion among virtual interlayer excitons.

Recently, optical Stark shifts were reported in both the *K*- and *K*′-valleys of WS_2_ for excitation above the A-exciton resonance and assigned to coupling between the A-exciton and intervalley biexcitons^54^. In that study, indirect evidence of a red-shift of the *K*-valley and a larger blue-shift of the *K*′-valley for *σ*^+^ excitation was inferred from two-color pump probe measurements. Such single-wavelength measurements can be misleading; see Supplementary Note 5. For example, the photo-induced absorption feature associated with coherent creation of biexcitons could be misinterpreted as evidence of an anomalous optical Stark shift; see Supplementary Fig. 6. Here we directly measure changes to the exciton absorption spectrum and do not observe evidence for optical Stark shifts associated with intervalley or intravalley biexcitons for photon energies above the exciton resonance. The bound biexciton contribution to the OSE is predicted only for photon energies red-shifted below the exciton resonance^34^, so no bound biexciton contributions are expected for photoexcitation above resonance. Bound intervalley biexciton contributions to the OSE were recently experimentally confirmed in MoSe_2_ for photon energies tuned just below the A-exciton resonance^44^.

It is noteworthy that the observed anomalous blue-shift of the A-exciton energy due to the valley-selective OSE remains present for linearly polarized pump and probe; see Supplementary Note 6 and Supplementary Fig. 7. Linearly polarized light, which is a superposition of *σ*^+^ and *σ*^−^ light, populates both the *K*- and *K*′-valleys. Since a blue-shift has been observed for co-circular polarization and no shift for cross-circular polarization, the blue-shift remains for collinearly polarized pump and probe. This shift is typically observed as a derivative-like feature in the photo-induced change in transmission (∆*T*/*T*_0_)^31,37^, which has previously been assigned to dynamic Coulomb screening^36^, and the resulting bandgap renormalization^29,30^. However, such screening-related spectral shifts should be relatively small and will persist for the entire lifetime of the charge carrier and exciton populations^31^. The blue-shift we observe is present only near time zero, while the pump pulse traverses the monolayer WS_2_. Therefore, this blue-shift is not consistent with dynamic screening. As discussed above, this shift is instead due to the valley-selective OSE.

We have examined the valley-selective optical Stark effect in monolayer WS_2_ for excitation wavelengths near the A-exciton resonance. We observe that a valley-selective blue-shift persists for above-resonance excitation. This effect is well isolated to a single valley, occurring in the *K* (*K*′) valley for *σ*^+^ (*σ*^−^) polarized light and can be observed via transient absorption spectroscopy for cocircularly polarized pump and probe pulses. This photo-induced blue-shift exhibits behavior consistent with coherent light−matter interactions and with a valley-selective optical Stark effect. The observed pump wavelength dependence is inconsistent with the two-level dressed-atom model that is typically employed to explain the optical Stark effect. Instead, our observations are consistent with an excitonic optical Stark effect model^33–35^ that includes exciton−exciton interactions in addition to the exciton−photon interactions. Here, we confirm the prediction from that theory that repulsion between virtual excitons will dominate near resonance and cause the optical Stark effect to produce a blue-shift even for pump photon energies tuned above resonance. This observation demonstrates the need to include many-body Coulomb effects, such as exciton−exciton interactions, when describing the optical Stark effect in low-dimensional semiconductors that support bound excitons. It may also draw into question predictions of band-inversion based on the two-level dressed-atom model. We also observe that coherent intervalley biexciton formation occurs for cross-circularly polarized pump and probe pulses, and proceeds via induced absorption of a probe photon in the opposite valley from the one initially excited. The sequential absorption of cross-circularly polarized pulses, the first tuned to the A-exciton resonance and the second detuned below it by the biexciton binding energy, represents an efficient means of preparing intervalley biexcitons states in monolayer transition metal dichalcogenides semiconductors for further study. A similar technique was recently used to create and then probe the fine structure of biexcitons in monolayer WSe_2_^55^. Such biexciton states are of interest for use as possible entangled photon sources^56^ and quantum logic gates^57^.

## Methods

### CVD growth of WS_2_ monolayers

Monolayer WS_2_ on SiO_2_/Si (275 nm thickness of SiO_2_) was prepared by chemical vapor deposition in a 2″ tube furnace. WO_3_ powder and sulfur precursors are heated to 825 °C under a 65 sccm argon and 10 sccm hydrogen flow. Perylene-3,4,9,10-tetracarboxylic acid tetrapotassium salt is used as seed molecules to promote lateral growth. This procedure produces large (>20 µm) grain uniform coverage monolayer WS_2_ that exhibits enhanced photoluminescence (PL)^58^. After growth, the WS_2_ films were transferred onto 1-mm-thick double-side polished fused silica substrates using a wet transfer technique^59^. The monolayer nature of the films was confirmed by Raman and PL mapping. The PL spectrum is dominated by the A-exciton emission with no significant trion character^28^.

### Transient absorption spectroscopy

We use a standard transient absorption spectroscopy setup that has been described elsewhere^60^. Monolayer WS_2_ films were photoexcited using tunable ~150 fs pulses from an optical parametric amplifier focused to 2.22 ± 0.7 mm, with power levels between 100 and 800 µW corresponding to 5–20 µJ/cm^2^ and maximum absorbed fluences between 3.9 × 10^11^–3.1 × 10^12^ photons/cm^2^ at 610 nm. Excited state dynamics were probed with white-light continuum pulses that were derived from 1 kHz 150 fs 800 nm pulses from a Ti:Sapphire amplifier that were focused into a sapphire plate and were analyzed using a scanning monochromator. This white-light probe was sufficiently stable as to not require referencing for additional noise reduction. To eliminate artifacts in the TA spectra due to scatter from the excitation beam, simultaneous modulation of the pump and probe beams with a mechanical chopper was employed with lock-in detection at the sum of the two modulation frequencies. Polarizers were used to purify both pump and probe polarization; superachromatic quarter wave plates were used to create right- and left- hand circularly polarized pulses. The circularly polarized white light transmitted through the sample was rotated back to linear polarization prior to entering the monochromator via a superachromatic quarter wave plate. The absorption spectrum at each time delay was calculated from the measured normalized change in transmission, *∆T/T*_0_^31^. Films were kept under dry air flow at room temperature during all measurements unless otherwise noted.

## Supplementary information

Supplementary Information

## Data Availability

The data that support the findings of this study are available from the corresponding author upon reasonable request.
