# Supplementary material for: Resonant optical Stark effect in monolayer WS2
Source: Nat Commun. 2019 Dec 5;10:5539. doi: 10.1038/s41467-019-13501-x (PMC6895111; doi:10.1038/s41467-019-13501-x)
Supplement: Supplementary file 1 — Supplementary Information [file 41467_2019_13501_MOESM1_ESM.pdf]

Supplementary Information: Resonant Optical Stark Effect in Monolayer WS_2

Paul D. Cunningham,[1,*] *Aubrey T. Hanbicki,*[1,2] *Thomas L. Reinecke,*[1] *Kathleen M. McCreary,*[1] *Berend T. Jonker*[1]

[1]U.S. Naval Research Laboratory, 4555 Overlook Avenue SW, Washington, DC 20375, United States

[2]Pressent Address: Laboratory for Physical Science, University of Maryland, 8050 Greenmead Drive, College Park, MD 20740, United States

*paul.cunningham@nrl.navy.mil

Supplementary Note 1. Intervalley biexciton photoinduced absorption

Supplementary Figure 1 shows that for cross-circular polarizations ($\sigma^-\sigma^+$), a new photoinduced absorption band appears below the A-exciton resonance, at 631 nm. We assign the new absorption feature to the coherent creation of intervalley biexcitons. The σ- pump photon creates an exciton in the K'-valley. The subsequent σ+ probe photon can then access the $|x_{K'}\rangle \rightarrow |x_{K'}x_K\rangle$ transition, which we observe as a photoinduced absorption band. Based on the separation between the A-exciton peak and this feature, we estimate the biexciton binding energy as 48 ± 5 meV.

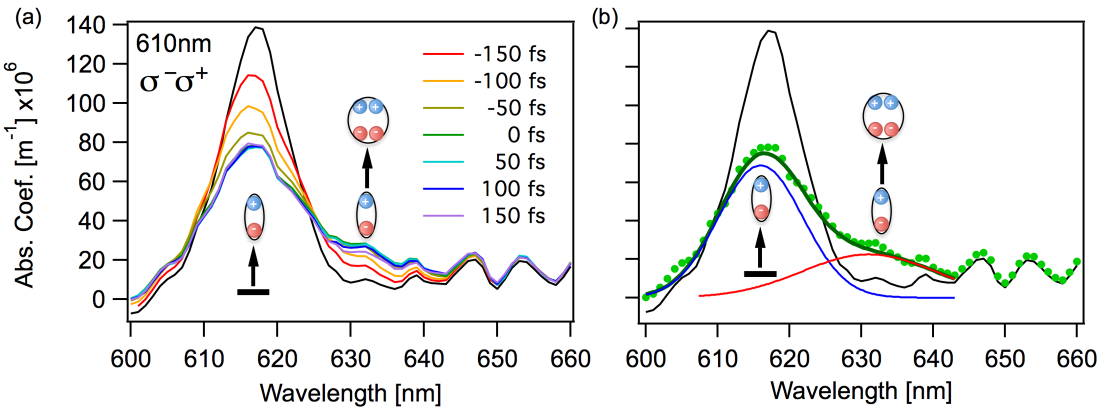

Supplementary Figure 1. Intervalley biexciton generation. (a) Absorption spectra at select time delays during 610 nm 10 μJ/cm^2 cross-circularly ($\sigma^-\sigma^+$) polarized photoexcitation of monolayer WS_2. The ground state to exciton absorption, and exciton to intervalley biexciton photoinduced absorption features are indicated. (b) The absorption spectrum for t = 0 fs (dots) and best fit (green) to the sum of two Gaussians, the first located at the exciton absorption of 616 nm (blue) and the second indicates the intervalley biexciton photoinduced absorption feature at 631 nm (red). The ground state absorption spectrum is included (black) for reference.

Supplementary Note 2. Intervalley charged biexciton and trion binding energies

The valley selective optical Stark effect in monolayer WS_2 can also be observed at low temperature. Here, the narrower absorption linewidths may make it easier to quantify small optical Stark shifts. Supplementary Figure 2 shows the valley-resolved TA spectra in monolayer WS_2 at 78K for a photoexcitation wavelength of 596 nm, which is resonant with the A-exciton. Unfortunately, illumination of WS_2 in vacuum leads to unavoidable creation of a ground state-to-trion absorption feature at 608 nm, shown in Supplementary Figure 3a, which clutters the spectra and complicates analysis. This trion absorption arises due to laser-induced removal of physisorbed oxygen from sulfur vacancies in WS_2, as previously reported by Currie et al.[1] Based on the separation between the A-exciton and trion features, we estimate the trion binding energy as 41 ± 5 meV. For cross-circular polarizations ($\sigma^{-}\sigma^{+}$), a new photoinduced absorption band associated with a multiple particle complex, shown in Supplementary Figure 3b, is just evident at 613 nm. This is analogous to the intervalley biexciton feature observed at room temperature. We estimate the binding energy of this multiple particle complex of 59 ± 5 meV, which is larger than the biexciton binding energy observed at room temperature, and indicates that the 78K induced absorption may originate from a charged biexciton, i.e. an exciton-trion complex.[2] This is consistent with the more trionic character of the absorption spectrum.

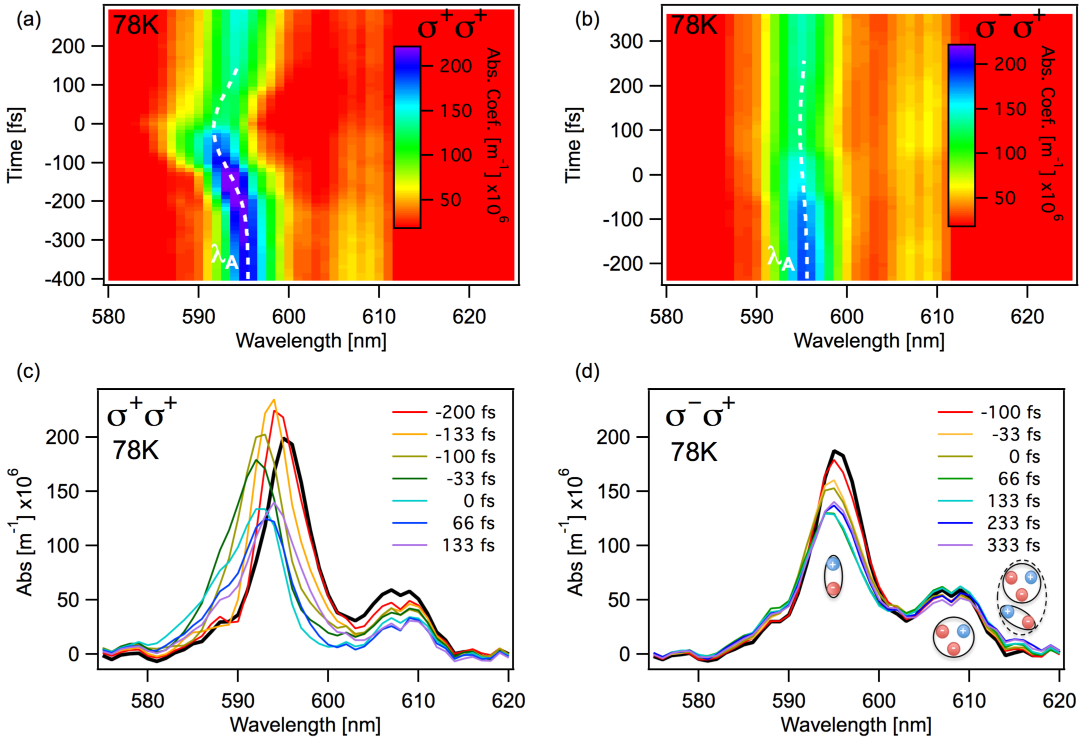

Supplementary Figure 2. Valley-selective optical Stark effect at 78K. Time dependent absorption spectrum of monolayer WS_2 recorded at 78K for 596 nm excitation with (a) co- ($\sigma^+\sigma^+$) and (b) cross-circularly ($\sigma^-\sigma^+$) polarization. The center wavelength of the A exciton resonance (λ_A) is indicated with a dashed white light. Time zero indicates when the pump and probe pulse are temporally overlapped. (c-d) Show absorption spectra at select time delays. In (d) the exciton absorption, trion absorption, and suspected intervalley charged biexciton photoinduced absorption features are indicated. The ground state absorption spectrum is included (black) for reference.

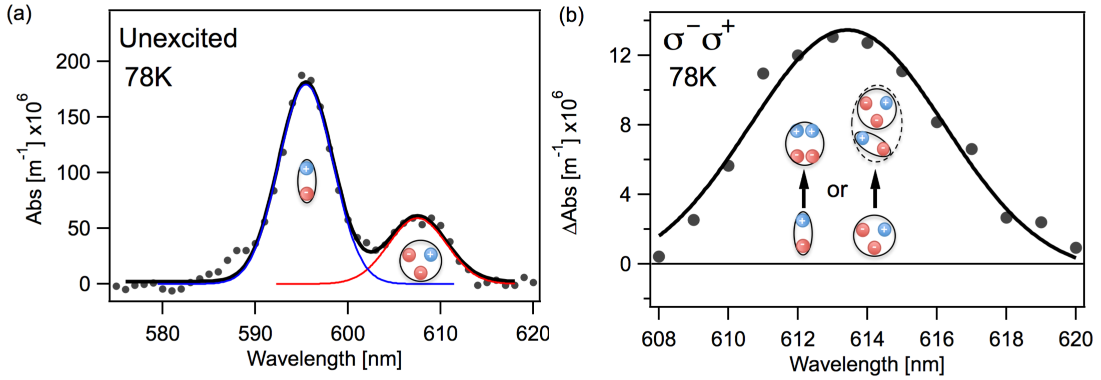

Supplementary Figure 3. Trions and Biexcitons at 78K. (a) Absorption spectrum of monolayer WS_2 recorded at 78K (dots) and best fit (black) to the sum of two Gaussians, the first located at the exciton absorption of 596 nm (blue) and the second located at the trion absorption feature of 608 nm (red). (b) Photoinduced change in absorption for 596 nm excitation with cross-circularly ($\sigma^-\sigma^+$) polarization at t = 0 fs (dots) and best fit to a Gaussian (black) indicating the suspected intervalley charged biexciton photoinduced absorption feature at 613 nm.

Supplementary Note 3. Fitting procedure for time-dependent absorption spectra

To quantify the size of the optical Stark shifts, we fit the time-dependent absorption spectra at each time delay with a Gaussian peak. Supplementary Figure 4 shows a representative example of this fitting procedure. Horizontal cuts through the time-dependent absorption spectra yields the absorption spectrum at each time delay. Each absorption spectrum is fit with a Gaussian peak. The time evolution of the center wavelength of that Gaussian yields the position of the A-exciton resonance as a function of time. From this, we estimate the maximum optical Stark shift for a given wavelength, pump power density, and polarization. Note that a small residual blue shift (~ 0.75 nm or

2.5 meV) persists after the coherent optical Stark shift. This residual is due to the imbalance between bandgap renormalization and the exciton binding energy reduction associated with dynamic screening by the real exciton population.[3] Because this effect reaches its maximum ~ 1ps after the OSE, its contribution to the measured optical Stark shifts is taken as negligible.

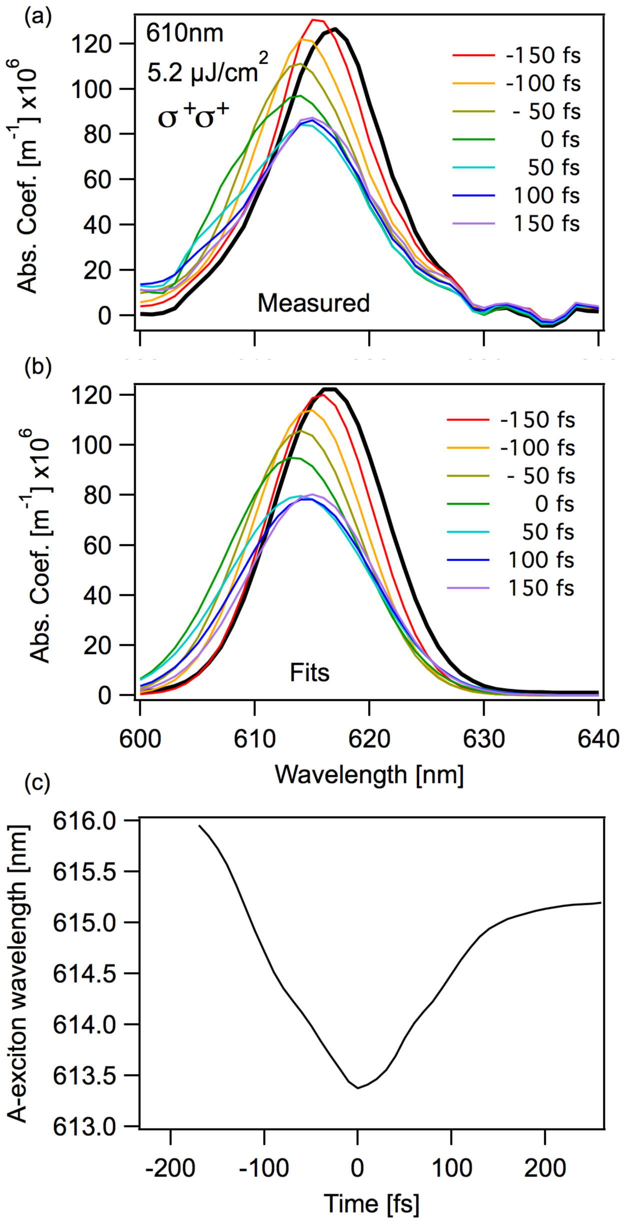

Supplementary Figure 4. Gaussian fits to time-dependent Absorption data. (a) Absorption spectra at select time delays during 610 nm 5.2 μJ/cm^2 co-circularly ($\sigma^+\sigma^+$) polarized photoexcitation of monolayer WS_2. (b) Gaussian fits to the measurements in (a). The ground state absorption spectrum is included (black) for reference. (c) The center wavelength of the A-exciton absorption as a function of time derived from the fits in (b).

Supplementary Note 4. Excitation wavelength dependence of the OSE

We used transient absorption spectroscopy to measure the valley-selective OSE in monolayer WS_2 as a function of excitation wavelength. Supplementary Figure 5 shows the time-dependent absorption spectra measured for both co- and cross-circular polarizations for various pump wavelengths. Pump pulses were tuned in energy both above and below the A-exciton resonance while the pulse width and power density were held approximately constant at 150 fs and 10 μJ/cm^2 respectively. The maximum photo-induced shifts were estimated by fitting each time step of the time-dependent absorption spectra with a Gaussian peak as described in the preceding section.

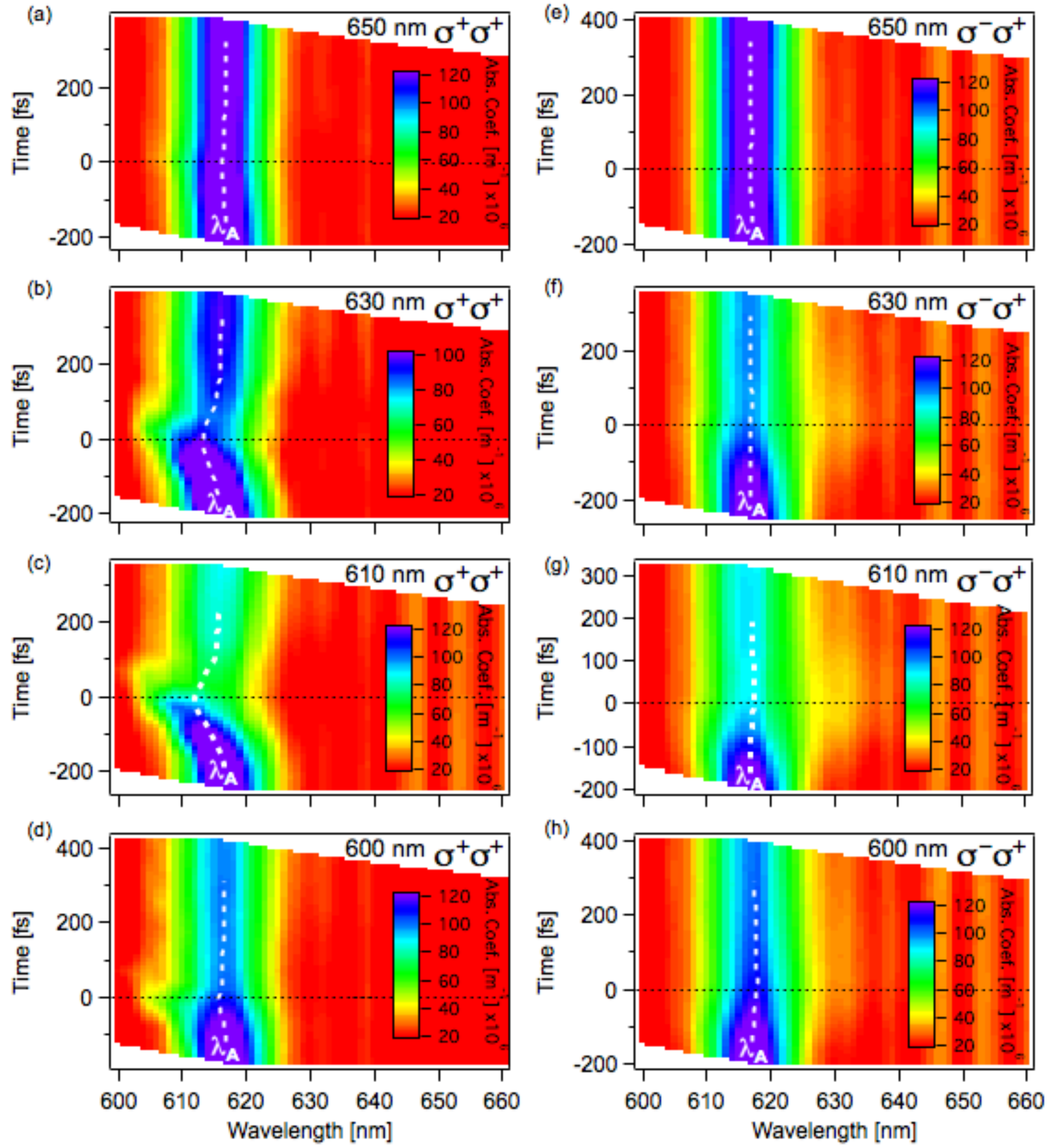

Supplementary Figure 5. Valley-selective optical Stark effect measurements. Time dependent absorption spectrum of monolayer WS_2 for (a-e) co- ($\sigma^+\sigma^+$) and (e-h) cross-circularly polarized ($\sigma^-\sigma^+$) light for pump excitation wavelengths of (a,e) 650 nm, (b, f) 630 nm, (c, g) 610 nm, and (d, h) 600 nm. The center wavelength of the A exciton resonance (λ_A) is indicated with a dashed white light. Time zero indicates when the pump and probe pulse are temporally overlapped.

Supplementary Note 5. Single wavelength OSE measurements

Here, we demonstrate that single wavelength, i.e. two-color pump probe, measurements of the OSE can be misleading, yielding artificial results. Supplementary Figure 6 summarizes photoinduced changes in transmission for 610 nm excitation and probed at a wavelength of 635 nm, which is just below the exciton resonance. The large decrease in transmission, i.e. increase in absorption, for cross-circularly polarized ($\sigma^-\sigma^+$) pulses may cause the observer to conclude that a very large red-shift is present. On the other hand, the small increase in transmission, i.e. decrease in absorption, for co-circularly polarized ($\sigma^+\sigma^+$) pulses may cause the observer to conclude that a small blue-shift occurs. The time dependence of this effect yields a large polarization centered at time zero, consistent with a coherent process driven by light-matter coupling. The surprising sub-linear power dependence seems to suggest that the observed effect goes beyond the typical OSE described by the two-level dressed exciton model. These observations are qualitatively similar, though opposite in direction, to recent reports of anomalous optical Stark shifts attributed to coupling between excitons and intervalley biexcitons.[4] However, the full transient absorption spectra we have shown, e.g. in the preceding section, show conclusively that a large red-shift is not present for cross-circularly polarized ($\sigma^-\sigma^+$) pulses. Instead, the increased absorption at 635 nm is caused by coherent creation of intervalley biexcitons, as detailed above and in the main text. Similarly, we have shown that a large blue-shift occurs for co-circularly polarized ($\sigma^+\sigma^+$) pulses, and that its fluence dependence is approximately linear with power. We therefore conclude that single wavelength measurements of the OSE can be misleading.

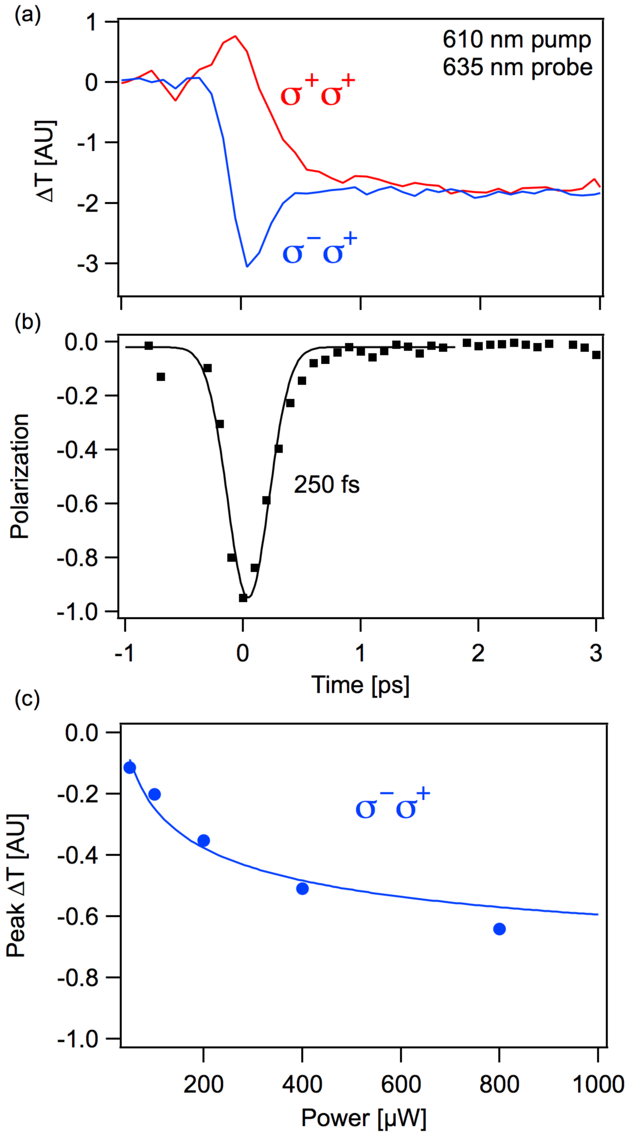

Supplementary Figure 6. Single-wavelength pump-probe measurements. (a) Single wavelength transient absorption dynamics in monolayer WS_2 measured for 610 nm excitation and 635 nm probe for (red) co- ($\sigma^+\sigma^+$) and (blue) cross-circularly polarized ($\sigma^-\sigma^+$) light. (b) The computed polarization from the measurements in (a). (c) The power dependence of the peak change in transmission (ΔT) measured for cross-circularly polarized ($\sigma^-\sigma^+$) light.

Supplementary Note 6. OSE observed with linearly polarized light

The valley-selective OSE remains present for linearly polarized pump and probe. Supplementary Figure 7 shows the time-dependent photoinduced changes in transmission, and corresponding time-dependent absorption spectra, for resonant excitation of monolayer WS_2 with collinear polarization. Because linearly polarized light is a superposition of σ^+ and σ^- polarizations, we expect it to experience changes to both the K- and K'-valleys. We have shown above that a blue-shift occurs for co-circular polarization and no shift occurs for cross-circular polarization. Therefore, both the K- and K'-valleys should blue-shift in response to a linearly polarized pump pulse and those shifts can be measured with a linearly polarized probe. This is indeed what we observe, as shown in Supplementary Figure 7.

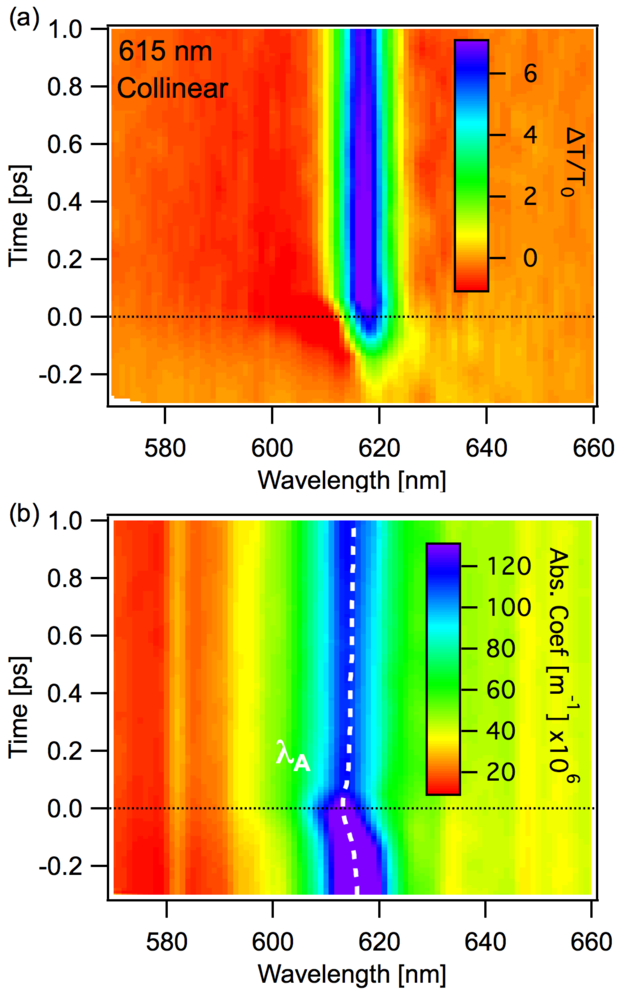

Supplementary Figure 7. Optical Stark effect with linearly polarized light. (a) Photo-induced change in transmission and (b) corresponding time-dependent absorption coefficient of monolayer WS_2 measured for collinearly polarized 615 nm excitation pulses. Time zero indicates when the pump and probe pulse are temporally overlapped. In (b) the center wavelength of the A exciton resonance (λ_A) is indicated with a dashed white light.

REFERENCES

1. Currie, M.; Hanbicki, A. T.; Kioseoglou, G.; Jonker, B. T. Optical control of charged exciton states in tungsten disulfide. *Appl. Phys. Lett.* **106,** 201907 (2015).

2. Paur, M.; Molina-Mendoza, A. J.; Bratschitsch, R.; Watanabe, K.; Taniguchi, T.; Mueller, T. Electroluminescence from multi-particle exciton complexes in transition metal dichalcogenide semiconductors. *Nat. Commun.* **10,** 1709 (2019).
3. Cunningham, P. D.; Hanbicki, A. T.; McCreary, K. M.; Jonker, B. T. Photoinduced Bandgap Renormalization and Exciton Binding Energy Reduction in WS_2. *ACS Nano* **11,** 12601-12608 (2017).
4. Sie, E. J.; Lui, C. H.; Lee, Y.-H.; Kong, J.; Gedik, N. Observation of Intervalley Biexcitonic Optical Stark Effect in Monolayer WS_2. *Nano Lett.* **16,** 7421-7426 (2016).
